# Mitochondria-associated non-coding RNAs and their impact on drug resistance

**DOI:** 10.3389/fphar.2025.1472804

**Published:** 2025-02-26

**Authors:** Xingna An, Lina Sun, Huan Zheng, Yinghui Xiao, Weixia Sun, Dehai Yu

**Affiliations:** ^1^ Department of Core Facility, The First Hospital of Jilin University, Changchun, Jilin, China; ^2^ Department of Hematology-Oncology, Affiliated Hospital of Changchun University of Traditional Chinese Medicine, Changchun, Jilin, China; ^3^ Department of Nephrology, The First Hospital of Jilin University, Changchun, Jilin, China

**Keywords:** mitochondria, microRNA, lncRNA, drug resistance, cancer

## Abstract

Drug resistance is a prevalent challenge in clinical disease treatment, often leading to disease relapse and poor prognosis. Therefore, it is crucial to gain a deeper understanding of the molecular mechanisms underlying drug resistance and to develop targeted strategies for its effective prevention and management. Mitochondria, as vital energy-producing organelles within cells, have been recognized as key regulators of drug sensitivity. Processes such as mitochondrial fission, fusion, mitophagy, changes in membrane potential, reactive oxygen species (ROS) accumulation, and oxidative phosphorylation (OXPHOS) are all linked to drug sensitivity. Non-coding RNAs (ncRNAs) enriched in mitochondria (mtncRNA), whether transcribed from mitochondrial DNA (mtDNA) or from the nucleus and transported to mitochondria, can regulate the transcription and translation of mtDNA, thus influencing mitochondrial function, including mitochondrial substance exchange and energy metabolism. This, in turn, directly or indirectly affects cellular sensitivity to drugs. This review summarizes the types of mtncRNAs associated with drug resistance and the molecular mechanisms regulating drug resistance. Our aim is to provide insights and strategies for overcoming drug resistance by modulating mtncRNAs.

## Introduction

Drug resistance occurs when a patient becomes less sensitive to a drug. Generally, prolonged use of a drug often leads to the development of drug resistance, resulting in reduced drug efficacy (Hughes and Andersson, 2015). Drug resistance poses a major obstacle to effective treatment, as most patients may develop resistance to a certain drug during therapy. To achieve better therapeutic efficacy, patients often need to switch drugs to continue treatment (Nikolaou et al., 2018).

Drug resistance can be classified into two types: primary and acquired drug resistance (Lampis et al., 2020). Primary drug resistance is defined as insensitivity to a drug at the onset of treatment, often resulting from genetic mutation, epigenetic modulation, or adaptation (Shah and Rawal, 2019; Heng et al., 2019; Wein and Loi, 2017). For instance, icotinib, an EGFR-tyrosine kinase inhibitor (TKI), is used to treat non-small-cell lung cancer (NSCLC) with epidermal growth factor receptor (EGFR) mutation (Zhong et al., 2017). In EGFR-sensitive mutant adenocarcinoma, primary drug resistance is defined as patients treated with icotinib whose disease does not improve, and instead worsens within 3 months (Zhong et al., 2017). On the other hand, acquired drug resistance is characterized by a decrease or loss of cellular sensitivity to a drug after prolonged exposure (Menchon, 2015). It encompasses various mechanisms, such as the inhibition of cell death (apoptosis suppression), altered expression of drug transporters, changes in drug metabolism, epigenetic modifications affecting drug targets, enhanced DNA repair, and gene amplification (Zhai et al., 2014; Corbett et al., 2020; Lu et al., 2018; Liu et al., 2020a; Ponnusamy et al., 2020). For instance, patients with lung adenocarcinoma (LUAD) who are initially treated with afatinib (a second-generation EGFR-TKI) may exhibit partial response or stable disease (Yamaoka et al., 2017). However, some patients develop resistance to afatinib after 6 months, with nearly half of those acquiring resistance showing EGFR-T790M mutation (Wu et al., 2016). Acquired drug resistance is also linked to mitochondrial metabolism. For instance, vemurafenib, a B-Raf proto-oncogene (BRAF) inhibitor, is used to treat melanoma caused by BRAF mutation (Bollag et al., 2012). Studies have revealed that melanoma patients undergoing vemurafenib treatment experience relapse within 6–7 months, attributed to the upregulation of anaplerotic mitochondrial metabolism and the acquisition of a survival advantage under nutrient-depleted conditions (Delgado-Goni et al., 2020).

The mitochondrion is a critical organelle in eukaryotic cells, primarily responsible for oxidation-reduction reactions and ATP synthesis (Annesley and Fisher, 2019). Mitochondria possess their own DNA, known as mitochondrial DNA (mtDNA), which encodes 13 proteins, most of which are involved in OXPHOS (Annesley and Fisher, 2019). Other proteins within mitochondria are encoded by nuclear genes and then imported into the organelle (Annesley and Fisher, 2019). Mitochondria are involved in various cellular processes, including cell stress responses, immune reactions, signal transduction, apoptosis, and calcium homeostasis (Anderson et al., 2019). Mutations in mtDNA can lead to mitochondrial dysfunction, potentially resulting in drug resistance (Molnar and Kovacs, 2017). For instance, Ni et al. demonstrated that mutations in mitochondrial genes of ovarian cancer cells can diminish respiratory chain activity and remodel cancer cell metabolism, leading to platinum resistance (Ni et al., 2020). Similarly, Lee et al. observed that depletion of mtDNA in colon cancer cells reduces ATP production and increases the expression of multidrug resistance 1 (MDR1) and P-glycoprotein, thereby conferring resistance to paclitaxel (Lee et al., 2008).

Noncoding RNAs (ncRNAs) are RNA molecules that do not encode proteins (Wei et al., 2020). Based on their length, ncRNAs are classified into small ncRNAs (sncRNAs, 18∼200 nt) and long ncRNAs (lncRNAs, >200 nt) (Wei et al., 2020). NcRNAs play essential roles in various cellular functions, including cell proliferation, autophagy, apoptosis, and the pathogenesis of diseases and drug resistance (Wei et al., 2020; Wang et al., 2019a). These molecules are found not only in the cytoplasm, nucleus, and matrix but also within mitochondria (Liu and Shan, 2021). Mitochondrial ncRNAs comprise two types: those encoded by mtDNA and those encoded by nuclear DNA and subsequently translocated into mitochondria (Kim et al., 2017). NcRNAs contribute to the regulation of mitochondrial dynamics, metabolism, ROS production, membrane potential, and overall cellular function. For instance, Zhou et al. screened 2175 mitochondria-related lncRNAs, among which 13 could accurately and independently predict the prognosis of LUAD patients (Zhou et al., 2023). Das et al. reported that miR-18c, a nuclear DNA-encoded miRNA, is translocated into mitochondria, where it inhibits the translation of mt-COX1, leading to the remodeling of complex IV and increased ROS production in rat cardiac myocytes (Das et al., 2012). The interplay between ncRNAs and mitochondria also plays a crucial role in drug resistance. For example, overexpression of miR-27a increases mitochondrial ROS levels by attenuating nuclear factor erythroid-2-related factor 2 (Nrf2) signaling pathway and the glutathione synthesis in MCF-7 cells, rendering them more sensitive to paclitaxel or doxorubicin (DOX), two conventional chemotherapy drugs for breast cancer (Ueda et al., 2020). Additionally, upregulation of miR-193a-3p can modulate serine/arginine-rich splicing factor 2 (SRSF2) expression, inhibit mitochondrial-dependent cellular apoptosis, and confer resistance to cisplatin in gastric cancer cells (Lee et al., 2019).

NcRNAs play a crucial role in regulating cellular behavior, including drug resistance, through the modulation of mitochondrial function, as evidenced by numerous studies. To date, there have been no reports on the association of mitochondria-related ncRNAs with drug resistance. In this review, we comprehensively examine published research papers available on PubMed (https://pubmed.ncbi.nlm.nih.gov) to summarize the types and functions of mtncRNAs. Specifically, we explore the correlation between these mtncRNAs and drug resistance, highlighting the underlying molecular mechanisms. Our goal is to offer insights and strategies for enhancing drug resistance by modulating mtncRNAs.

## Mitochondria and drug resistance

**FIGURE 1:**
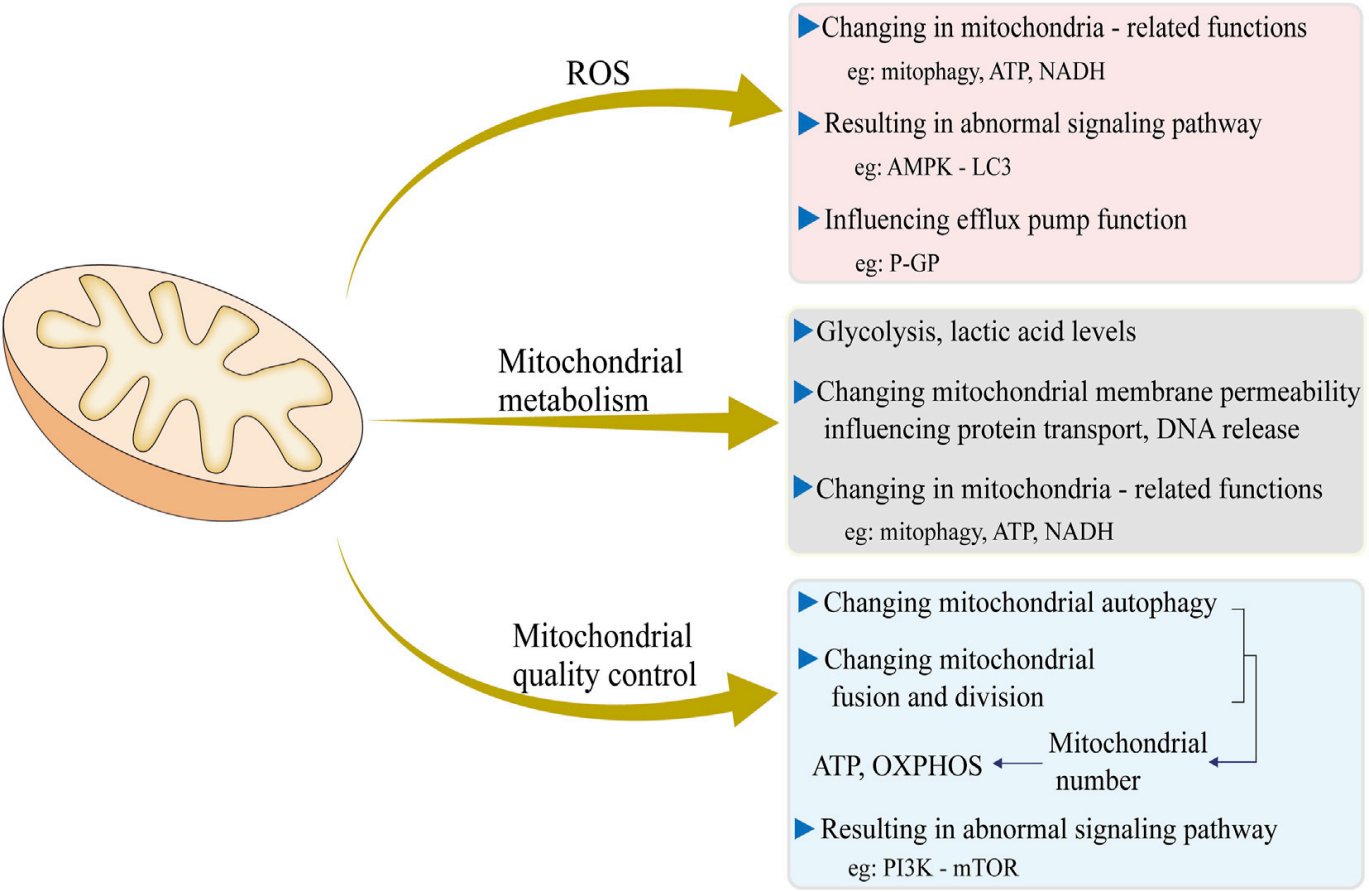
Three major mitochondrial events, including ROS production, mitochondrial metabolism, and mitochondrial quality control, and their impact on cell drug resistance and the underlying molecular mechanisms.

Mitochondria are crucial for fundamental cellular processes. They generate up to 95% of a cell’s energy (ATP) through OXPHOS, which powers various cellular activities (Tzameli, 2012). Moreover, mitochondria play key roles in calcium buffering and the regulation of apoptosis (Chandhok et al., 2018). Many drugs, particularly anti-cancer medications, target molecules within these essential processes to induce cell apoptosis (Okon and Zou, 2015). As a result, any alterations in mitochondrial function-such as changes in ROS production, metabolic processes, and mitochondrial dynamics-can significantly affect a cell’s sensitivity to these drugs (Figure 1).

### Mitochondrial ROS and drug resistance

ROS, generated as a byproduct of mitochondrial energy production, serve as signaling molecules within the physiological range (Kasai et al., 2023). ROS include free radicals such as superoxide, hydroxyl radicals, and singlet oxygen, as well as non-radical species like hydrogen peroxide, which is formed through the partial reduction of oxygen (Zhang et al., 2016). Under normal physiological conditions, cells maintain redox homeostasis—a balance between oxidants and antioxidants (Li et al., 2023). Abnormal ROS production has been linked to various diseases, including cancer, chronic obstructive pulmonary disease and Alzheimer’s disease (Fairley et al., 2023; Yu et al., 2024). Drug-resistant cancer cells exhibit distinct ROS levels compared to their normal counterparts (Patel et al., 2020). Currently, there are two conflicting viewpoints regarding the correlation between ROS levels and drug resistance. One perspective holds that ROS levels are diminished in drug-resistant cells. For instance, Gao et al. reported that cisplatin-resistant muscle-invasive bladder cancer cells (e.g., UMUC3, T24, and J82) exhibited higher autophagic flux and lower ROS levels in mitochondria. They indicated that compared to normal bladder cancer cell lines, cisplatin-resistant muscle-invasive bladder cancer cells show increased expression of calcium binding protein 39 (CAB39), causing activation of liver kinase B1 (LKB1), adenosine monophosphate-activated protein inase (AMPK), and microtubule-associated protein one light chain 3 (LC3), which makes the cells more likely to survive (Gao et al., 2023). Similarly, Xu et al. found that in sorafenib-resistant hepatocellular carcinoma (HCC) cells, peroxisome proliferator-activated receptor γ coactivator 1β (PGC1β) degrades, leading to ROS decline and thereby protecting mitochondrial function and integrity, all of which contribute to development of resistance (Xu et al., 2021). Given the observed reduction in ROS levels in drug-resistant cells, increasing ROS production is considered a strategy to overcome drug resistance. Wang et al. utilized lipid membrane-coated silica-carbon hybrid nanoparticles and near-infrared (NIR, 800 nm) laser irradiation to generate ROS in tumor cell mitochondria. This system oxidized nicotinamide adenine dinucleotide (NADH), reduced ATP production and impaired efflux pump function, ultimately overcoming multidrug resistance (Wang et al., 2018). In NSCLC, chloroquine-an autophagy inhibitor-has been shown to inhibit autophagy through upregulating the key autophagy protein P62, thereby increasing ROS production, accumulating damaged mitochondria and enhancing A549 cell sensitivity to paclitaxel (Datta et al., 2019). Conversely, the other contends that in drug-resistant cells, ROS levels are elevated and the resistance can be overcome by reducing ROS. In gefitinib-resistant lung cancer cells, ROS levels increased, accompanied by decreased OXPHOS and ATP (Okon et al., 2015). Moreover, Okon et al. found that the use of antioxidants could reduce the production of ROS, subsequently weakening drug resistance. The same ROS and mitochondrial alterations were found in MCF-7 breast cancer cells (Mahalingaiah and Singh, 2014).

### Mitochondrial metabolism and drug resistance

Mitochondria, as the central metabolic hubs of cells, primarily generate energy by utilizing glycolytic substrates. However, an imbalance in mitochondrial metabolism can lead to detrimental effects on cells. For example, while normal mammalian cells predominantly generate energy through OXPHOS, cancer cells rely more heavily on glycolysis, a phenomenon referred to as the Warburg effect (Bhattacharya et al., 2016). This shift in metabolic pathways modifies the sensitivity of tumor cells to anti-cancer drugs. Arata et al. found that tumor cells can evade apoptosis by depending on glycolysis rather than OXPHOS, which leads to drug resistance (Tomiyama et al., 2006).

The family with sequence similarity 83B (FAM83B) is highly expressed in lung adenocarcinoma and serves as a poor prognosis marker. Overexpression of FAM83B inhibits the translocation of calbindin 2 (CALB2) from the cytoplasm to mitochondria. As a result, mitochondrial mass, ATP production, and mitochondrial membrane potential increase, which in turn reduces chemotherapy-induced apoptosis (Wang et al., 2024).

Mitomycin C (MMC)-based chemotherapy is a mainstay treatment for non-muscle-invasive bladder cancer (NMIBC). Nevertheless, up to 65% of NMIBC cases develop resistance to MMC (Tomasz, 1995; Sylvester et al., 2006). Oresta et al. discovered that drug-resistant NMIBC displays hyperglycolysis and metabolic reprogramming after chemotherapy. This leads to decreased mitochondrial permeability and impaired mtDNA release, thereby failing to activate the inflammasome and affecting immunogenic cell death. Additionally, low mitochondrial complex I abundance was observed in NMIBC patients who relapsed after MMC treatment (Oresta et al., 2021).

Elevated lactic acid levels signify abnormal mitochondrial metabolism (Marcucci and Rumio, 2021). Suk et al. reported that in sorafenib-resistant HCC cells, both glycolysis and lactate production are upregulated. They proposed that treatment with the ketone body D-β- hydroxybutyrate (β-HB) may inhibit glycolytic-lactate metabolism and suppress the B-raf/mitogen-activated protein kinase (MAPK) pathway and the mesenchymal N-cadherin-vimentin axis. This, in turn, may reverse sorafenib resistance in HCC (Suk et al., 2023).

### Mitochondrial quality control and drug resistance

Common mechanisms of mitochondrial quality control include mitochondrial dynamics, mitochondrial autophagy (mitophagy), mitochondrial protein homeostasis, and mitochondrial DNA damage repair. Mitophagy involves the selective clearance of mitochondria, typically removing abnormal or damaged mitochondria, thereby promoting cell survival and development (Lu et al., 2013). In normal cells, mitophagy maintains equilibrium, but this balance is disrupted in drug-resistant cells. For instance, in HCC treated with cisplatin, resistance frequently develops (Verslype et al., 2012). Sheng et al. elucidated the connection between cisplatin resistance in HCC and mitochondrial quality control. They discovered that cisplatin enhances lysosome synthesis in HCC cells, promoting mitochondrial-lysosome crosstalk and facilitating the clearance of damaged mitochondria (Sheng et al., 2019). Moreover, increased mitochondrial autophagy was found to confer greater cisplatin resistance, and the use of phosphatidylinositide 3-kinase (PI3K)/mammalian target of rapamycin (mTOR) inhibitors can disrupt this crosstalk and increase the sensitivity of HCC cells to cisplatin (Sheng et al., 2019). Clinically, efforts have been made to counteract drug resistance by inhibiting mitochondrial autophagy. For example, Chang et al. demonstrated that in recurrent drug-resistant ovarian cancer, utilizing nano-drug ^188^Re-Liposome inhibited mitochondrial autophagy, thereby overcoming resistance in ovarian cancer (Chang et al., 2017).

Mitochondrial division and fusion are essential for cells to eliminate damaged mitochondria while retaining healthy ones (van der Bliek et al., 2013). In normal cells, the dynamic balance of mitochondrial division and fusion is tightly regulated. However, in drug-resistant cells, this balance is often disrupted. ROS are frequently implicated as a significant factor contributing to this imbalance. For instance, Han et al. observed that hypoxia-induced ROS trigger mitochondrial fission and cisplatin resistance through downregulation of phosphorylated dynamin-related protein 1 (p-Drp1, Ser637) and mitofusin 1 (Mfn1) in ovarian cancer cells (Han et al., 2019). Similarly, Wu et al. reported that in head and neck squamous cell carcinoma, hypoxia enhances ROS release and promotes mitochondrial fission factor (Mff) expression and chemical sensitivity to cisplatin via hypoxia inducible factor 1 alpha (HIF1α)/Mff regulation (Wu et al., 2021). Similar to ROS, the relationship between mitochondrial division and drug resistance is also different in different tumors. Moreover, abnormal expression of key factors regulating mitochondrial fission, such as Drp1 and Mff, has been associated with drug resistance. It has been reported that in gemcitabine-resistant pancreatic cancer cell lines, upregulated Drp1 expression and increased mitochondrial number are accompanied by an increase in OXPHOS, ultimately leading to drug resistance (Masuo et al., 2023). Furthermore, in cisplatin-resistant HCC tissues, elevated Mff expression leads to increased Drp1 expression and promotes cisplatin resistance (Li et al., 2022).

### Other mechanisms

Studies have shown that ROS and ATP influence drug sensitivity by regulating drug efflux. P-glycoprotein (P-GP) plays a key role in drug efflux, and its upregulation reduces intracellular drug concentration, thereby diminishing the drugs’ cytotoxic effects and contributing to drug resistance (Kim et al., 2024). Hyperthermia boosts the levels of ROS and P-GP in prostate tumor. However, certain drugs, such as 4-(2-aminoethyl)benzenesulfonyl fluoride (AEBSF) can reduce ROS production and lower P-GP expression, leading to decreased drug efflux and enhanced drug sensitivity (Wartenberg et al., 2005). Guo et al. developed a nanomedicine combining paclitaxel, polyethylene glycol, oxidized sodium alginate, and functionalized graphene oxide, which generates excessive ROS and inhibits P-GP’s efflux function by targeting the mitochondrial respiratory chain. This enhances intracellular drug concentration and increases drug sensitivity in paclitaxel (PTX)-resistant gastric cancer (Guo et al., 2021).

Abnormal DNA damage repair is a key factor contributing to drug resistance in tumors. One-carbon units, such as amino acids and nucleotides, play a crucial role in DNA repair. Mitochondria supply the majority of these carbon units through serine catabolism (Ducker et al., 2016). In 5-FU-resistant colorectal cancer (CRC) cells, serine hydroxymethyltransferase-2 (SHMT2), an enzyme involved in mitochondrial serine metabolism, is significantly upregulated, leading to the production of large amounts of one-carbon units, particularly purines. The increased purine levels enhance DNA damage repair, thereby reducing 5-FU’s ability to induce DNA damage and diminishing the drug’s efficacy (Pranzini et al., 2022). Additionally, dihydroorotate dehydrogenase (DHODH) which is involved in pyrimidine synthesis and located in the inner mitochondrial membrane, has also been found to promote DNA damage repair and contribute to drug resistance in 5-FU-resistant CRC cells (Dong et al., 2024).

Table 1 summarizes the correlation between changes in mitochondrial function and drug resistance, particularly in tumor cells.

**TABLE 1:** Correlation between changes in mitochondrial function and drug resistance.

Disease	Mitochondrial function change	Resistant to	References
Bladder cancer	Mitochondria ROS ↓	Cisplatin	Gao et al. (2023)
HCC	Mitochondria ROS ↓	Sorafenib	Xu et al. (2021)
Bladder cancer	Glycolysis ↑	Mitomycin C	Sylvester et al. (2006), Oresta et al. (2021)
HCC	Glycolysis ↑	Sorafenib	Suk et al. (2023)
HCC	Lactic acid production ↑	Sorafenib	Suk et al. (2023)
HCC	Mitophagy ↑	Cisplatin	Verslype et al. (2012), Sheng et al. (2019)
Ovarian cancer	Mitophagy ↑	^188^Re-Liposome (a nano-drug)	Chang et al. (2017)
Ovarian cancer	Mitochondrial fission ↑	Cisplatin	Han et al. (2019)
HNSCC	Mitochondrial fission ↓	Cisplatin	Wu et al. (2021)
Pancreatic cancer	Mitochondrial fission ↑	Gemcitabine	Masuo et al. (2023)
HCC	Mitochondrial fission ↑	Cisplatin	Li et al. (2022)

## Mitochondria-related ncRNAs and drug resistance

NcRNAs constitute 97% of the RNA in mammalian cells (Liu et al., 2021). They are classified into several types, including microRNA (miRNA), lncRNA, circular RNA (cirRNA), and small nucleolar RNA (snoRNA) (Wang et al., 2019b). ncRNAs play crucial roles in regulating various biological processes, and their dysregulation is implicated in numerous diseases (Li et al., 2014; Jiang et al., 2020a; Xie et al., 2016). Research has demonstrated that ncRNAs can influence cell sensitivity to drugs by regulating mitochondrial functions, such as ROS production, ATP content, membrane potential, and mitochondrial dynamics.

### ncRNAs and drug resistance

MiRNA is the main class of small ncRNA, typically ∼22 nucleotides in length (Tasdelen and Sen, 2021). miRNAs regulate a wide range of cellular behaviors, including differentiation, proliferation, and apoptosis (Zhuo et al., 2013; Li et al., 2019; Singh et al., 2013), and they play pivotal roles in modulating drug sensitivity. It is generally accepted that miRNAs induce drug resistance through several mechanisms:

a) Modulation of apoptosis: miRNAs can regulate cell apoptosis, leading to drug resistance. For instance, Meng et al. showed that long-term 5-FU therapy downregulates miR-206 and upregulates its target protein Bcl-2 in CRC cells, suppressing apoptosis and increasing resistance to 5-FU (Meng and Fu, 2018). Similarly, miR-141-3p directly targets p53. Overexpression of miR-141-3p reduces p53 protein expression, thereby suppressing apoptosis and making glioblastoma cells resistant to temozolomide (TMZ) (Zhou et al., 2017).
b) Modulation of DNA damage repair: miRNAs can affect DNA damage repair processes, leading to drug resistance. Bortezomib treats multiple myeloma (MM) by inhibiting DNA repair. Yuan et al. reported that downregulation of miRNA-520g/h enhances DNA repair by increasing the expression of human apurinic/apyrimidinic endonuclease 1 (APE1), a DNA repair-related protein, inducing resistance to bortezomib in MM cells (Neri et al., 2011; Yuan et al., 2019). Xu et al. found that upregulation of miR-33b-3p facilitates DNA damage repair, leading to lung cancer cells’ resistance to cisplatin (Xu et al., 2016).
c) Regulation of autophagy: miRNAs can regulate autophagy, contributing to drug resistance. Autophagy involves the elimination of protein aggregates and damaged organelles through the lysosomal degradation pathway (Levine and Kroemer, 2008). Drug-induced autophagy can lead to the death of pathogenic cells and achieve therapeutic effects. Xu et al. reported that in MM cells, upregulation of miR-221/222 downregulates its target gene, autophagy-related gene 12 (*ATG12*), reducing phagocytosis and cell death, ultimately leading to dexamethasone resistance (Xu et al., 2019). Additionally, downregulation of miRNA-125b has been shown to enhance autophagy and reverse HCC cells’ resistance to oxaliplatin (Ren et al., 2018).

LncRNAs constitute another important class of ncRNAs, typically longer than 200 nucleotides (Derrien et al., 2012). They regulate gene expression through interactions with proteins, RNA, and DNA (Xing et al., 2021). Similar to miRNAs, lncRNAs participate in diverse cellular processes and also influence cell sensitivity to drugs (Kitagawa et al., 2013; Yuan et al., 2014; Sirey et al., 2019; Ballarino et al., 2016). LncRNAs mediate drug resistance through various molecular mechanisms:

a) Sponge effect on miRNAs: lncRNAs can act as sponges for miRNAs, leading to drug resistance. For instance, Shu et al. demonstrated that increased expression of LINC00680 in HCC targets miR-568, reducing its levels. This derepression activates protein kinase B 3 (AKT3), enhancing HCC stemness properties which reduces sensitivity to 5-FU chemotherapy (Shu et al., 2021). In breast cancer cells, lncRNA CYTOR binds to miR-125-5p, downregulating its levels and promoting the expression of serum response factor (SRF), thereby increasing resistance to tamoxifen (TAM) (Liu et al., 2020b).
b) Regulation of gene expression via epigenetic modifications: lncRNAs can regulate gene expression through epigenetic modifications such as DNA methylation, histone modification, chromatin remodeling, and RNA interference (Tammen et al., 2013). For example, downregulation of lncRNA HOTAIR in esophageal cancer cells decreases DNA methylation of the methylenetetrahydrofolate reductase (MTHFR) promoter, enhancing chemosensitivity to 5-FU (Zhang et al., 2020a). Silencing of LINC00673 in prostate cancer cells demethylates the kruppel-like factor 4 (*KLF4*) gene promoter, inhibiting proliferation and reducing resistance to paclitaxel (Jiang et al., 2020b). Zhu et al. reported that overexpression of lncRNA NEAT1 remodels chromatin and increases histone acetylation levels, promoting 5-FU resistance and enhancing cancer stemness (Zhu et al., 2020). In HCC, upregulation of lncRNA colorectal neoplasia differentially expressed (CRNDE) increases histone acetylation and upregulates EGFR expression, contributing to resistance against sorafenib (Liu et al., 2022).

### Mitochondria-related ncRNAs

ncRNAs are found not only in the cytoplasm but also within mitochondria (Liu and Shan, 2021). RNAs in mitochondria, known as mitochondrial RNA (mitoRNA), are encoded by mitochondrial DNA and transported from the cytoplasm (Kim et al., 2017). MitoRNAs play essential roles in regulating mitochondrial functions.

#### Mitochondria-related microRNA and transport

Many miRNAs are encoded by nuclear DNA and subsequently transported into mitochondria, such as let-7b, miR-302a, miR-365, miR-2392, and miR-125b (Barrey et al., 2011). Due to the double-membrane structure of mitochondria, some mitoRNAs must traverse the inner membrane to reach the mitochondrial matrix and exert their regulatory functions (Macgregor-Das and Das, 2018).

Like other miRNAs, mitomiRs also undergo a maturation process before being transported into mitochondria, as extensively reviewed (Macgregor-Das and Das, 2018; Han et al., 2006; Carthew and Sontheimer, 2009). The general process includes the following steps:

a) Transcription of the non-coding region of nuclear DNA to form a primary miRNA transcript (pri-miRNA).
b) Processing of pri-miRNA to generate precursor miRNA (pre-miRNA).
c) Export of pre-miRNA from the nucleus to the cytoplasm.
d) Splicing of pre-miRNA by Dicer to produce mature double-stranded miRNA, with one strand being degraded. In the cytoplasm, miRNA binds to the argonaute2 (Ago2) protein to form the RNA-induced silencing complex (RISC), facilitating gene regulation (Lian et al., 2009; Schirle and MacRae, 2012).

Free ago2: miRNA complex the RISC enters the inner mitochondrial membrane space through three main pathways:

a) Sorting and assembly machinery component 50 homolog (SAM50) or translocase of outer mitochondrial membrane 20 (TOM20) pathway: the Ago2: miRNA complex first passes through SAM50 or TOM20 on the mitochondrial outer membrane, and then enters the mitochondrial matrix through translocase of inner mitochondrial membrane (TIM) on the inner membrane (Macgregor-Das and Das, 2018).
b) PNPase-assisted entry: polynucleotide phosphorylase (PNPase), a protein located in the inner mitochondrial membrane extending into the intermembrane space, assists the Ago2:miRNA complex in entering the mitochondrial matrix via the SAM50/TOM20/TIM pathway (Jusic et al., 2020).
c) Mitochondrial targeting sequences: miRNAs with mitochondrial targeting sequences (UGUCGGUGAGU) at their 3′-end can bind to Ago2 and translocate into mitochondria through the SAM50/TOM20/TIM entry gates. Examples include miR-181a and miR-181c (Jusic et al., 2020).

#### Mitochondria-related lncRNAs and transport

Similar to microRNAs, mitochondria-related lncRNAs (mito-lncRNAs) are encoded by both mitochondrial and nuclear genes. LncRNAs are involved in the regulation of mitochondrial function, including mitochondrial metabolism and apoptosis. However, the mechanism by which nucleus-encoded lncRNAs are transported to mitochondria remains unclear.

### Mitochondria-related ncRNAs and drug resistance

**FIGURE 2:**
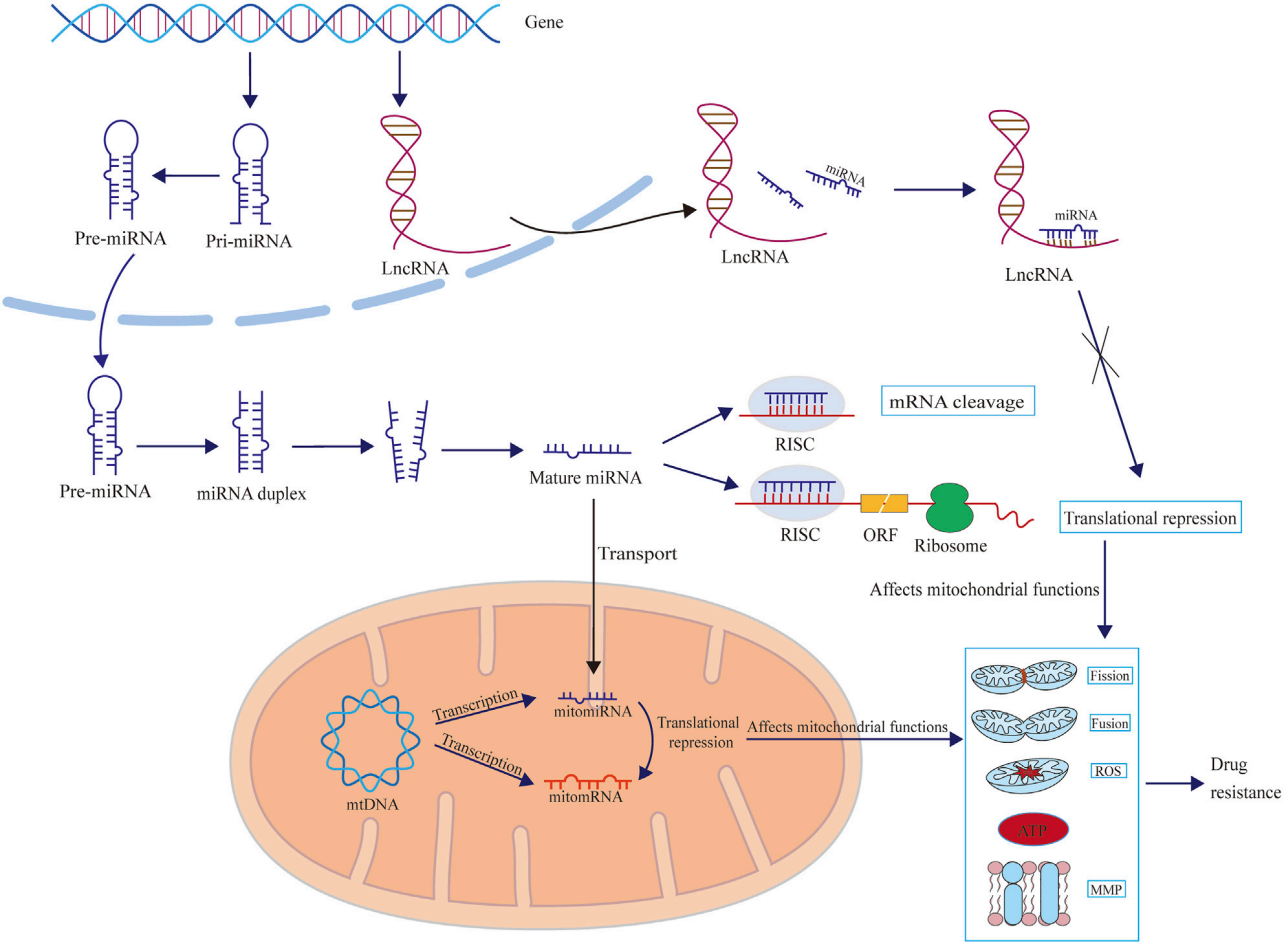
The schematic diagram illustrates how microRNAs and lncRNAs influence drug resistance by regulating mitochondrial functions, including mitochondrial fission, fusion, ROS and ATP production, and mitochondrial membrane potential (MMP). Mitochondria-related microRNAs can exert regulatory effects in the cytoplasm (mainly by affecting the synthesis of mitochondria-related proteins) and can also be transported into the mitochondria to exert regulatory functions (mainly by affecting the synthesis of enzymes related to redox reactions). However, there are currently no reports showing how lncRNAs are transported to the mitochondria and exert regulatory functions.

In drug-resistant cells, several changes occur in mitochondrial function, such as increased production of mitochondrial ROS, abnormal mitochondrial fission, and loss of mitochondrial membrane potential (ΔΨm). Certain mitochondria-related ncRNAs can regulate mitochondrial function both inside and outside the mitochondria, thereby affecting drug resistance (Figure 2) (Song et al., 2013; Roh et al., 2016; Shen et al., 2013; Kulkarni et al., 2016; Liang et al., 2021).

#### MitomiRs and drug resistance

Accumulating research indicates a close correlation between drug sensitivity and mitochondrial function, which can be modulated by mitochondrial ncRNAs. Fan et al. reported that mitomiR-2392, a microRNA that translocates from the nucleus to mitochondria, was upregulated in cisplatin-resistant tongue squamous cell carcinoma (TSCC) cells (Fan et al., 2019). The elevated expression of mitomiR-2392 leads to a decrease in OXPHOS and an increase in glycolysis. This metabolic reprogramming enables TSCC cells to develop resistance to cisplatin (Fan et al., 2019). In addition, in TSCC, miR-485-5p was found to inhibit mitochondrial division and reduce cisplatin sensitivity by targeting the mitochondrial fission protein (FIS1) (Fan et al., 2015). Furthermore, in cisplatin-resistant TSCC, the expression of mitomiR-5787 was reported to be downregulated. This downregulation triggers a shift from OXPHOS to aerobic glycolysis, accompanied by a reduction in both MT-CO3 and ATP levels, thus further enhancing drug resistance. Moreover, it has been verified that the expression of mitomiR-5787 and MT-CO3 is indeed decreased in TSCC patients with a poor prognosis (Chen et al., 2019).

Variations in mitochondria-related miRNAs in breast cancer can significantly alter mitochondrial function and affect drug sensitivity. In DOX-resistant MCF-7 cells, the decreased expression of miR-125b correlates with reduced mitochondrial damage and ROS production (Hu et al., 2018). Yuan et al. discovered that in DOX-resistant MCF-7 cells, the reduction in miR-133a is accompanied by an increase in uncoupling proteins (UCP)-2 (Yuan et al., 2015). UCPs are a subset of mitochondrial anion transporters that influence ATP synthesis and mitochondrial ROS generation (Brand and Esteves, 2005). Given the connection between abnormal ROS/ATP levels and drug resistance in breast cancer cells (Liu et al., 2019; Fujiwara-Tani et al., 2022), it is plausible that variations in miRNA-induced ROS generation and ATP production play pivotal roles in altering cancer cells’ drug sensitivity.

In HCC, hypoxia induces the upregulation of miR-210-5p and downregulation of its target ATPase family AAA domain-containing 3A (ATAD3A), which inhibits mitophagy and promotes sorafenib resistance (Wu et al., 2020). Jeong et al. identified that overexpression of miR-24 in SK-Hep1 human hepatoma cells reduces H2AX, a mitochondrial outer membrane protein, disrupting mitochondrial ATP content, membrane potential, and oxygen consumption, ultimately leading to defects in the insulin signaling pathway and insulin resistance (Jeong et al., 2017). In sorafenib-resistant HCC cells, overexpression of miR-30a-5p inhibits glycolysis and increases apoptosis, thereby enhancing sensitivity to sorafenib. Researchers also demonstrated that systemic delivery of a cholesterol-modified Ago:miR-30a-5p complex significantly suppresses tumor growth in mice harboring sorafenib-resistant HCC tumors (Zhang et al., 2020b). Lu et al. reported that in HCC, the knockdown of miR-3689a-3p increased the expression of the copper chaperone for superoxide dismutase (CCS). The elevated CCS levels enhance superoxide dismutase type 1 (SOD1)-mediated scavenging of mitochondrial oxidative stress, rendering HCC insensitive to sorafenib (Lu et al., 2023).

Various strategies have been attempted to enhance drug sensitivity in glioblastoma multiforme (GBM). It has been demonstrated that hypoxia-induced upregulation of miR-26a strengthens resistance to TMZ by inhibiting mitochondrial apoptosis and preserving mitochondrial function (Ge et al., 2018). Tafazzin (TAZ), a protein located in the mitochondrial membrane involved in maintaining mitochondrial membrane potential, serves as a critical regulator of mitochondrial apoptosis. It has been shown that upregulation of miR-125 b reduces TAZ expression, thereby increasing the sensitivity of glioma cells to tumour necrosis factor (TNF)-related apoptosis-inducing ligand (TRAIL) by promoting mitochondrial apoptosis (Ma et al., 2019).

Besides the tumors mentioned above, mitomiRs have also been reported to be associated with drug resistance in other cancers. For example, inhibiting miR-98 in bladder cancer cells increases mitochondrial fusion and decrease mitochondrial membrane potential, making bladder cancer sensitive to cisplatin and DOX (Luan et al., 2018). Lee et al. reported that in gastric cancer cells, upregulation of miR-193a-3p increases anti-apoptotic genes such as Bcl-2 and decreases pro-apoptotic genes such as Bax, which enhances resistance to cisplatin by regulating mitochondria-related apoptotic pathways (Lee et al., 2019).

#### MitolncRNA and drug resistance

Some studies have also shown that lncRNAs can modulate drug sensitivity by influencing mitochondrial function. However, compared to microRNAs, research on this aspect of lncRNAs is still relatively scarce. A notable example is SAMMSON (Survival Associated Mitochondrial Melanoma-Specific Oncogenic Non-Coding RNA), which was initially discovered in melanoma. SAMMSON has been identified as a significant regulator of cancer cell metabolism through interaction with p32, a key regulator of mitochondrial homeostasis and metabolism (Leucci et al., 2016). Dewaele et al. demonstrated the interaction between lncRNA SAMMSON and p32 in uveal melanoma cells (UM) and found that inhibiting SAMMSON disrupts mitochondrial protein translation and impairs mitochondrial function, thereby inhibiting UM metastasis (Dewaele et al., 2022). Orre et al. demonstrated that silencing SAMMSON reduces DOX resistance in MCF-7 cells by decreasing mitochondrial ROS production and increasing mitochondrial viability (Orre et al., 2021). Additionally, Tian et al. reported that in TSCC, lncRNA MPRL (miRNA processing related lncRNA) binds to pre-miR-485-5p and affects the expression of the targeted protein FIS1. Overexpression of lncRNA MPRL reduces miR-485-5p levels and increases FIS1, which enhances sensitivity to cisplatin by promoting mitochondrial fission and apoptosis (Tian et al., 2019).

The abnormal expression of different ncRNAs have diverse effects on mitochondrial function. Table 2 shows how ncRNAs disrupt mitochondrial function and in turn contribute to the development of drug resistance.

**TABLE 2:** Mitochondria-related ncRNAs and regulation of mitochondrial function in cell drug resistance.

Mitochondria-related ncRNAs	Change	Diseases	Effects on mitochondria	Drug resistance	References
MicroRNA					
miR-2392	↑	TSCC	Decreasing OXPHOS; raising glycolysis	Cisplatin ↑	Fan et al. (2019)
miR-485-5P	↑	TSCC	Inhibiting mitochondrial division; reducing mitochondrial division proteins FIS1	Cisplatin ↑	Fan et al. (2015)
miR-5787	↓	TSCC	Inhibiting the translation of MT-CO3	Cisplatin ↑	Chen et al. (2019)
miR-125b	↓	Breast cancer	Decreasing mitochondrial damage and ROS production	Doxorubicin ↑	Hu et al. (2018)
miR-133a	↓	Breast cancer	Increasing UCP-2	Doxorubicin↑	Yuan et al. (2015)
miR-210-5p	↑	HCC	Downregulating ATAD3A; inhibiting mitophagy	Sorafenib ↑	Wu et al. (2020)
miR-30a-5p	↑	HCC/HCC tumors	Inhibiting glycolysis; increasing apoptosis	Sorafenib ↓	Zhang et al. (2020b)
miR-24	↑	Liver related diseases	Disrupting mitochondrial function, e.g., ATP content, membrane potential and oxygen consumption	Insulin ↑	Jeong et al. (2017)
miR-26a	↑	GBM	Inhibiting mitochondria apoptosis; protecting mitochondrial function	Tomozolomide ↑	Ge et al. (2018)
miR-125b	↓	Glioma	Inducing mitochondrial apoptosis	TRAIL ↑	Ma et al. (2019)
miR-98	↓	Bladder cancer	Inducing mitochondrial fusion; inhibiting mitochondrial membrane potential	Multidrug ↓	Luan et al. (2018)
miR-3689a-3p	↓	HCC	Scavenge mitochondrial oxidative stress	Sorafenib↑	Lu et al. (2023)
LncRNA					
LncRNA SAMMSON	↓	Breast cancer	Reducing mitochondrial ROS production; increasing mitochondrial viability	Doxorubicin ↓	Orre et al. (2021)
LncRNA SAMMSON	↓	Uveal melanoma cell (UM)	Affecting mitochondrial protein translation	Inhibiting UM metastasis	Dewaele et al. (2022)
HOTAIRM1	↑	Glioblastoma	Decreasing ROS level	Radioresistance ↑	Ahmadov et al. (2021)
lncRNA MPRL	↑	TSCC	Enhancing mitochondrial fission and apoptosis	Cisplatin ↓	Tian et al. (2019)

## Preclinical and clinical studies

Drugs targeting the modulation of mitochondrial function have been gaining attention in recent years, especially in the treatment of cancer, neurodegenerative diseases, and metabolic diseases. These drugs have emerged as potential therapeutic targets. For example, Elamipretide has been shown to increase ATP synthesis and improve muscle strength in the treatment of human hypertrophic cardiomyopathy; Bendavia reduced oxidative stress and improved renal function and structure in coronary artery stenosis (CAS) (Nollet et al., 2023; Sun et al., 2015). Clinical trials are currently evaluating its efficacy in acute coronary syndrome and neurodegenerative diseases. There are also mitochondria-targeting drugs such as valproic acid for the epilepsy, and Idebenone for hereditary optic neuropathy (LHON) and DOX-mediated cardiotoxicity (Kudin et al., 2017; Yu-Wai-Man et al., 2024; Qiu et al., 2024).

The combination of anticancer drugs and mitochondria-targeting drugs to regulate mitochondrial metabolism has emerged as a promising strategy to reverse drug resistance. In H460 xenografted mouse, IACS-010759, a clinical mitochondrial complex I inhibitor, in combination with trametinib, decreased carnitine palmitoyl transferase IA (CPTIA) expression and significantly reduced oxygen consumption rate (OCR) and OXPHOS, enhanced apoptosis, and reversed resistance to trametinib (Feng et al., 2023). In TAM-resistant breast cancer cells, both cellular and animal experiments have proven that baicalein in combination with TAM can limit glucose uptake, ATP production, and lactic acid production, thereby downregulating aerobic glycolysis, enhancing sensitivity to TAM and enhancing mitochondrial apoptotic pathways (Chen et al., 2021).

Currently, several drugs targeting ncRNAs are undergoing clinical studies. Some of these drugs are already in clinical trials, such as miR-21 (Medina et al., 2010; Ling et al., 2013). In HCC patients with high recurrence and poor prognosis, low miR-541 expression correlates with reduced sorafenib sensitivity. In nude mice, combining miR-541 with sorafenib synergistically downregulated autophagy-related genes, inhibiting tumor growth, volume, and weight. Upregulating miR-514 also enhanced sorafenib sensitivity in mice (Xu et al., 2020). Drugs targeting ncRNAs application to patients still face many challenges, especially the issue of side effects. Researchers used the miR-34 mimic MRX34 to treat patients with advanced solid tumors, but it caused severe immune-mediated toxicity leading to death in five patients in a phase I clinic (Hong et al., 2020). Overall, drug studies targeting ncRNAs with mitochondrial regulatory functions are still in the relatively early stages, and this could be another strategy to reverse drug resistance.

## Conclusion and perspectives

In theory, drug resistance can develop in the treatment of most diseases if administered for a sufficient duration. Numerous small molecule substances can influence drug sensitivity through diverse mechanisms, offering potential avenues to reverse drug resistance.

Mitochondria, as the cellular energy hubs, play integral roles in regulating various cellular functions. Increasing evidence underscores their involvement in modulating cell sensitivity to drugs. ncRNAs are closely associated with drug resistance, affecting pivotal cellular processes like proliferation, apoptosis, and metabolism. However, our understanding of how ncRNAs contribute to drug resistance through their influence on mitochondrial function is still in its early stages. There is limited research on the regulatory roles of ncRNAs that are synthesized in the nucleus and then transported to mitochondria. In particular, studies investigating the relationship between lncRNAs, mitochondrial function, and drug resistance are notably scarce. Recently, our research team has utilized high-throughput sequencing to identify alterations in mitochondrial ncRNA (including microRNAs, lncRNAs and circRNAs) expression profiles in drug-resistant tumor cells. We are currently conducting functional studies to elucidate the impact of these ncRNA abnormalities on mitochondrial function. These findings will be detailed in forthcoming publications.

This review summarizes the interplay between mitochondria and drug resistance, categorizes mitochondria-related ncRNAs, and explores how these ncRNAs influence cell drug sensitivity by modulating mitochondrial function. It is our hope that this review will stimulate further research into the regulatory roles of mitochondria-related ncRNAs in both mitochondrial and cellular functions.
